# The Impact of Design Factors on Drivers’ and Non-Drivers’ Comprehension of Variable Message Signs

**DOI:** 10.3390/bs15091288

**Published:** 2025-09-20

**Authors:** Ana Hernando, Antonio Lucas-Alba, Andrés S. Lombas, Maria Teresa Blanch

**Affiliations:** Emotion, Regulation and Adjustment Group, Department of Psychology and Sociology, University of Zaragoza, 50009 Zaragoza, Spain; anahdo@unizar.es (A.H.); slombas@unizar.es (A.S.L.); blanchmi@unizar.es (M.T.B.)

**Keywords:** design criteria, road users, traffic sign comprehension, variable message signs

## Abstract

This study examines differences in comprehension between drivers and non-drivers when interpreting variable message signs (VMSs) combining three elements—a pictogram, an arrow, and a city name—to indicate temporary traffic events (e.g., “congestion before Lyon”). A total of 101 participants (51 non-drivers) were shown VMS displays reporting an event associated with one of four cities and were asked to identify the event’s location (before or after the city). The experiment employed a mixed factorial design. Two between-subject factors were included: Driving License (present vs. absent) and Route Listing (present vs. absent). Four within-subject factors were manipulated: Complementary Message (present vs. absent), Landmark Order (bottom-up vs. top-down), Event Location (before vs. after), and Arrow Function (explicit vs. generic). The dependent variable was the accuracy of location identification. The results showed that, for drivers, the most effective combination was bottom-up order with an explicit arrow, followed by bottom-up with a generic arrow, and then top-down with an explicit arrow. For non-drivers, no significant differences were found between these combinations. However, comprehension decreased across both groups when the message used a top-down order and a generic arrow. Overall, the data suggest that the G1c template from the 1968 Convention is not effective for either group. Prior driving experience seems to favor one specific design, the bottom-up order with explicit arrow, while non-drivers perceive all functionally viable options—including that one—as equally valid.

## 1. Introduction

**Figure 1:**
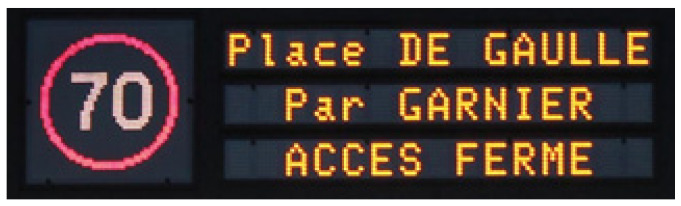
Example of a variable message sign (VMS) with two columns; one for pictogram and one for alphanumeric text (after Nouvier et al., 2009).

In the past five decades, global population and urbanization have both doubled, accompanied by an unprecedented growth in mobility through migration, tourism, and motor vehicle use (United Nations, 2024; United Nations, Department of Economic and Social Affairs, Population Division, 2024; International Energy Agency, 2023). This high density of movement underscores the importance of road signage, with the 1968 Convention on Road Signs and Signals serving as the most widely adopted standard (United Nations Economic Commission for Europe, 2006, 2025). Its universalist aim—to provide messages understandable across thousands of languages—remains crucial, especially in multilingual urban contexts (Eberhard et al., 2022; Pasikowska-Schnass, 2016; Von Ahn et al., 2010). However, while mobility technologies have advanced rapidly, updates to the Convention have been slow and limited, covering mainly fixed signs and offering little guidance for electronic signage such as variable message signs (VMS) (United Nations Economic Commission for Europe, 2006). Since the 1980s, telematics technologies have expanded road information possibilities, but have also led to de-harmonization across countries due to technical and economic constraints (COST 30 bis, 1985; Haitz & Tsao, 2011). As a result, by the late 20th and early 21st centuries, many European countries shared the same signaling devices (see Figure 1), but not necessarily the type or layout of the content they displayed, nor a shared international purpose (Blanch et al., 2011). To sum up, the international harmonization of complex signage—particularly that used in VMS—remains an open challenge, relevant not only to motorized drivers but to an increasingly mobile global population.

### 1.1. Understanding Complex Traffic Signs Entails Cognitive Reasoning Processes

**Figure 2:**
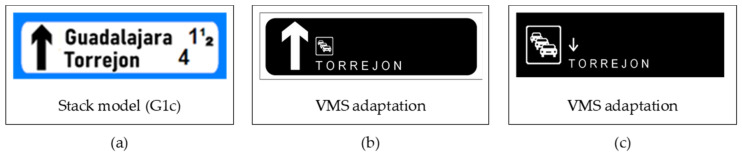
Examples of messages used in this study (Congestion before Torrejon) and their transformation from the original stack model to the VMS: (**a**) example of the original stack model-G1c from the 1968 Convention; (**b**) adaptation B (generic arrow); (**c**) adaptation C (explicit arrow).

Research on traffic signs has traditionally focused more on attentional and perceptual aspects than on comprehension (Castro & Horberry, 2004; Cristea & Delhomme, 2014; Reinolsmann et al., 2019; Shalloe et al., 2014). While drivers can easily recognize familiar elements such as congestion or roadwork icons stored in long-term memory (Crundall & Underwood, 2001; Lucas-Alba et al., 2011; Shinar & Vogelzang, 2013), comprehension of complex traffic messages requires active reasoning when these elements are combined in novel ways (Castro et al., 2008; Vargas et al., 2011). In such cases (see Figure 2b,c), drivers must construct meaning within seconds, going beyond mere retrieval from memory. This reasoning process can be explained by Mental Model Theory (Johnson-Laird, 1983, 2006, 2010), which views reasoning as a general cognitive function, and by Preferred Mental Model Theory, which emphasizes shared strategies and directional preferences in constructing mental representations (Ragni & Knauff, 2013; Jahn et al., 2007; Oberauer & Wilhelm, 2000; Taylor & Tversky, 1992).

### 1.2. Optimizing Infrastructure Use While Preserving International Standardization: Qualitative Location in VMS

Building on the design strategies found in the 1968 Convention sign catalog (e.g., diagrammatic vs. stacked formats) and the display capabilities of the most common variable message signs (VMS) in Europe (Ellenberg & Fabré, 1995; see Figure 1), various design factors have been explored to communicate the qualitative location of variable events (e.g., “congestion before Lyon”) without relying on a specific language. This has involved examining different combinations of three key elements: an iconic pictogram, a toponym, and an arrow.

Previous studies involving drivers from Sweden, the Netherlands, Italy, and Spain found that arranging the three elements on a VMS vertically in a bottom-up order led to higher comprehension rates than a horizontal (left-to-right) layout (Hernando et al., 2022a). Regarding the event location, the research also showed that messages indicating an event occurring before a location were better understood than those referring to the event after it. A subsequent study explored different vertical arrangements (with the landmark order being either bottom-up or top-down), comparing two viable VMS design options inspired by G1c-type stacked messages from the 1968 Convention (see Figure 2a). In Adaptation B (“generic arrow”), orientation was conveyed by an arrow placed in the main pictogram area. If the message is interpreted as a stacked message (G1c type), it would be read top-down, with the meaning: “Congestion in the direction of Torrejon.” (it is important to highlight that in Spain, fixed road signs follow the G1c model of the 1968 Convention, see Figure 2a). In Adaptation C (“explicit arrow”), the arrow points directly to the toponym. If the top-down orientation prevails, the interpretation is also “Congestion in the direction of Torrejon” (Hernando et al., 2022b).

The results revealed that comprehension varied considerably as a function of arrow type and landmark order. In the explicit-arrow messages, comprehension was good in both the top-down and bottom-up conditions; however, in the generic-arrow messages, good comprehension was observed only in the bottom-up condition. As in the study by Hernando et al. (2022a), comprehension of the qualitative location of an event before the town was better than in the after condition, and this pattern held across all combinations of reference point order (town) and arrow function (Hernando et al., 2022b).

### 1.3. Is the Understanding of Complex Signage Equally Accessible to Drivers and Non-Drivers?

In the field of road signage, previous research has primarily focused on motor vehicle drivers’ comprehension. In particular, Berrio et al. (2023) showed in their systematic review that 91.4% of the studies focused on motor vehicle drivers. However, the current traffic context involves multiple agents (motorists, motorcyclists and cyclists, pedestrians, etc.), and the actions of each are intertwined with those of others—especially in urban environments. The World Health Organization (2018) has pointed out that pedestrians, cyclists, and motorcyclists are generally overlooked in the design of the traffic system, despite accounting for more than half of all traffic fatalities. It is essential that all potential road users—not just motorists—are able to understand and respond appropriately to the information displayed on road signage (Maulina et al., 2022; Oh et al., 2013). Accordingly, an increasing number of studies are beginning to address the needs of these other user groups (Tekeş et al., 2021; Trifunović et al., 2017). In this broader context of inclusive mobility, it is worth noting that most signage systems in airports, metros, railways, museums, ports—and on roads—share common design features such as arrows and pictograms to guide movement (International Organization for Standardization, 2023; Harding et al., 2017; Sign Research Foundation, 2016; Majid & Ismail, 2016).

Are non-drivers poorer reasoners when faced with these configurations? An experienced driver possesses domain-specific knowledge related to road use, such as being able to interpret complex signs or anticipate the behavior of other vehicles. Does the comprehension of these signs, specifically, depend on domain-specific knowledge (Alexander, 1992; Chi et al., 1981) associated with driving? What differences can be observed between experts and novices? If the essential comprehension of certain signs relies on more general reasoning processes, this could facilitate their broader integration into road contexts. However, insofar as prior knowledge supports the generation of mental models in working memory, and non-drivers may lack such support, it can be predicted that they will be at a disadvantage. The processes of standardizing road signage (e.g., the Convention catalog) are slow and often exclude signs that are already in use—or could be. Moreover, making such signs more accessible to non-drivers could contribute to their safety, provided they are considered when such signage is displayed. It is important to note that this claim could not be made for certain traffic signs that are graphical but arbitrary (non-iconic; see Eco, 1996), such as the B3 sign “priority road” (a white square with a black rim, and in the center a smaller square in either yellow or orange, also with a black rim) in the 1968 Convention. In sum, exploring non-drivers’ comprehension of certain VMS makes sense under a dual rationale: first, because it suggests that the process of road sign standardization might be accelerated if non-drivers also understand them; and second, because the comprehension of certain messages is relevant in contexts beyond traffic alone, as event location matters not only on the road.

### 1.4. Goals of the Study

**Figure 3:**
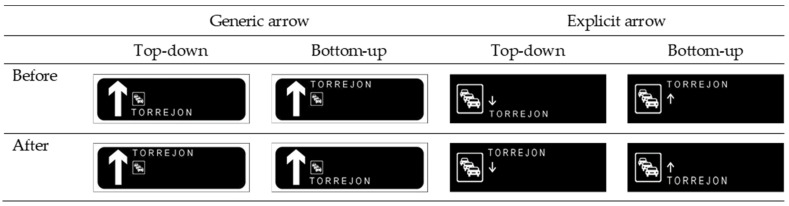
A stimulus example of designs combining the three factors examined regarding the format or content of the VMS messages.

This study explores the extent to which the comprehension of different design variants (see Figure 3) depends on knowledge acquired during driver training. The starting point is that the specific elements used in the configurations examined—a toponym, an arrow, and a congestion icon—are, in fact, within anyone’s grasp.

## 2. Materials and Methods

### 2.1. Participants

Data from 101 individuals (70 women) were analyzed, with a mean age of 23.64 years (*SD* = 4.98; *Min.* = 18; *Max.* = 51). Of this sample, 50 participants were included in the driver group (43 held a driver’s license and 7 had passed the theoretical driving exam), while 51 participants neither held a drivers’ license nor had passed the theoretical exam (non-driver group). The driver group was also included in the sample of a previous study, Hernando et al. (2022b), specifically those who responded to the messages with the elements justified to the left (see Section 2.2. Stimuli). The sample showed a high level of education (85% were university graduates or students, 9% had completed vocational training, and 6% held a secondary education diploma).

The driver group constituted 27 women and 23 men, and the mean age was 25.54 years (*SD* = 6.33; *Min.* = 21.50; *Max.* = 51). In terms of educational level, 41 were university graduates or students, 7 had completed vocational training, and 2 held a secondary education diploma. Regarding the driving experience, 8 participants did not have driving experience, 25 had less than 5 years, 16 had between 6 and 15 years, and only one person had more than 15 years of driving experience.

With respect to the non-driver group, it included 43 women and 8 men, with a mean age of 21.78 years *(SD* = 1.85; *Min.* = 18; *Max.* = 31). Regarding their level of education, 45 were university graduates or students, 2 had completed vocational training, and 4 held a secondary education diploma. None of them had driving experience.

### 2.2. Stimuli

MediaLab software (version 2014) was used to present the stimuli and record participants’ responses. The VMS displays occupied an area of 1024 × 290 pixels on the screen. Two VMS templates were adopted: one showed a 174 × 174-pixel pictogram plus a 60 × 70-pixel arrow, and the other one showed a 100 × 174-pixel arrow plus an 80 × 80-pixel pictogram. In both cases the text was Arial size 40 (see Figure 1). All displayed city names contained three syllables, and the pictograms were well-known, iconic representations (Lucas-Alba et al., 2011).

### 2.3. Design and Procedure

Participants were recruited via posters displayed around the university campus and provided written informed consent prior to participation. The study was conducted as part of a research project approved by the Research Ethics Committee of the Autonomous Community of Aragón (CEICA; reference PI20/076). Testing was conducted individually in a private university room. Participants were seated approximately 60 cm from a computer with a 22-inch monitor and were instructed to imagine driving along the A-2 highway from Guadalajara to Getafe, being informed that they would pass through three cities in that order: Alcala, Torrejon, and Coslada. They were randomly assigned to either a Route Listing group, in which they were requested to order the three city names to reinforce memory of the route sequence, or a Non-Route Listing group, which proceeded directly to the comprehension task. On each trial, two messages were presented sequentially: a complementary message followed by the target message. The aim was to evaluate whether the prior presentation of one adaptation of the VMS (see Figure 2b,c) affected—by improving or hindering—the comprehension of the other. Participants were told to respond as quickly and accurately as possible, basing their judgment exclusively on the second message. Two response alternatives (e.g., “Before Torrejón” vs. “After Torrejón”) appeared on the screen, with their order counterbalanced across trials, and responses were made using designated keys. After completing three practice trials, participants proceeded with 24 experimental trials presented in a randomized order, of which 16 were ultimately included in the statistical analyses. All stimuli were justified to the left. Each trial followed this sequence: a fixation cross (500 ms), a complementary message or neutral cue (2 s), another fixation cross (500 ms), the target message (4 s). After this second message, the question (Where is the event?) together with the two possible answers (e.g., Before Torrejon, After Torrejon) was presented and remained visible until participants responded. Subsequently, a black screen appeared (3 s) between successive experimental trials. The session lasted approximately 20 min.

The experiment employed a mixed factorial design. The dependent measure was the number of correct responses in the VMS comprehension task. Two between-subject factors were included: Driving License (present vs. absent), indicating whether participants possessed a valid driving license, and Route Listing (present vs. absent), reflecting the presence or absence of a sequential listing of route destinations within the message. Four within-subject factors were manipulated across trials. Complementary message (present vs. absent) specified whether a preceding, informative display appeared before the target message; when absent, the initial display was replaced with a fixation cross. Landmark order (top-down vs. bottom-up) referred to the vertical arrangement of city names. The top-down strategy follows the stack model (left-to-right, top-to-down; Bergen & Chan, 2005; Spalek & Hammad, 2005), such that cities are listed from nearest (at the top) to the farthest (at the bottom). In contrast, bottom-up strategy simulates the spatial flow of the route, with the nearest element placed at the bottom of the message, and the farthest at the top (Fuhrman & Boroditsky, 2010; Rinaldi et al., 2018). Event location (before vs. after) indicated whether the traffic event described in the message occurred before or after a specified reference landmark. Originally, this factor also included messages that located the event between two cities. However, the present study focuses on the conditions involving a single landmark (before/after) on the VMS. Arrow function (generic vs. explicit) referred to the iconography and layout of the event indicator. In the generic design, the event pictogram was incorporated within the alphanumeric column while the upward arrow remained on the left; in the explicit design, the event pictogram was placed on the left, and a small arrow was positioned alongside the city names within the alphanumeric column. Each participant received all combinations of the within-subject manipulations in randomized order (see Appendix A).

## 3. Results

Statistical analyses were conducted using IBM SPSS software v.26. First, an analysis of variance (ANOVA) was conducted on message comprehension to examine the effects of arrow function (explicit/generic), complementary message (present/absent), landmark order (top-down/bottom-up), event location (before/after), route listing (present/absent), and driving license (present/absent). Since the primary objective of this study was to compare message comprehension between drivers and non-drivers, only the results involving the driving license factor are presented below.

Results showed that the main effect of driving license was not statistically significant *F*(1,97) = 0.32, *p* = 0.57, *η_p_*^2^ = 0.003. However, the interaction between landmark order and driving license had a significant effect, *F*(1,97) = 5.24, *p* = 0.024, *η_p_*^2^ = 0.051. Similarly, the interaction between landmark order, arrow function, route listing and driving license also had a significant effect, *F*(1,97) = 4.93, *p* = 0.029, *η_p_*^2^ = 0.048. Since the higher-order interaction incorporated all main effects and lower-order interactions, the analysis focused exclusively on this final interaction. This interaction was analyzed through a series of simple effect analyses, with Bonferroni correction applied to adjust for multiple comparisons.

**Figure 4:**
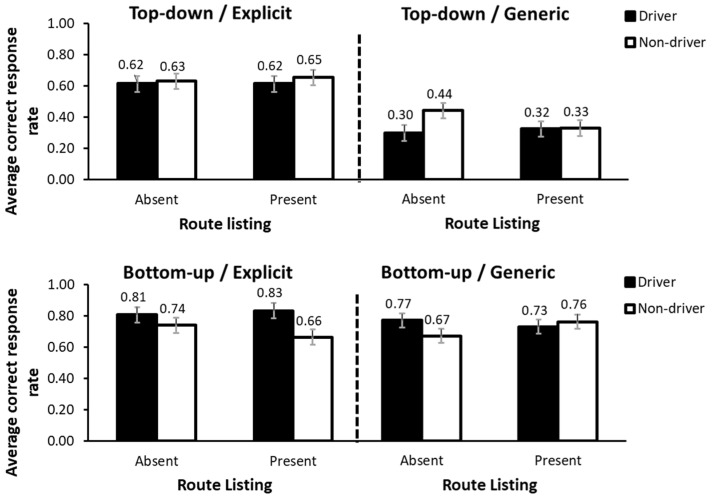
Response accuracy for the interaction between the factor combination (landmark order and arrow function), route listing and driving license. Error bars show the standard error of the mean.

First, a simple effect analysis was conducted to assess comprehension when the route listing was present compared to absent, while keeping the other factors constant. Results did not show statistically significant differences in any of the conditions (*p*s > 0.05). Second, a simple effect analysis was performed in which two of the factors were combined into a single factor with four levels. Specifically, the landmark order and arrow function factors were combined to create a new factor called combination, which included the following four levels: top-down order with generic arrow; top-down order with explicit arrow; bottom-up order with generic arrow; and bottom-up order with explicit arrow. This strategy was chosen because a graphical representation of the results (see Figure 4) indicated that, for each condition, the pattern of results was similar across the different combinations of these factors. After that transformation, a simple effect analysis was conducted to compare the different combinations of the factors, while keeping the remaining conditions constant. Results showed that the combination top-down/generic yielded the lowest rates of comprehension when the rest of variables remain constant (*p*s < 0.03). An exception emerged in the non-driver group when the route listing was absent, where comprehension in this combination did not significantly differ from top-down/explicit combination. Overall, the mean difference between the lowest-performing combination (i.e., top-down/generic combination) and the top-down/explicit combination was approximately 30%, while the differences from the other two combinations were around 40%. Results also showed similar comprehension rates for the bottom-up/explicit combination and the bottom-up/generic combination in both the driver and non-driver groups, regardless of route listing condition (mean difference of 7% and 1%, respectively; *p*s > 0.05). However, while drivers showed higher comprehension in the bottom-up/explicit combination compared to both the top-down/explicit (mean difference of 20%) and bottom-up/generic combinations (mean difference of 51%), non-drivers differed significantly only from the former combination (mean difference of 34%).

**Figure 5:**
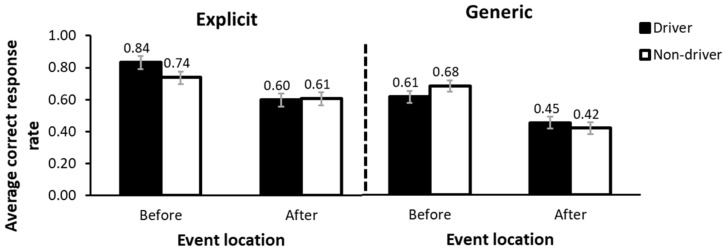
Response accuracy for the interaction between event location, arrow function and driving license. Error bars show the standard error of the mean.

The ANOVA conducted also showed a significant interaction between arrow function, event location and driving license, *F*(1,97) = 5.48, *p* = 0.021, *η_p_*^2^ = 0.053 (see Figure 5). As in the previous case, and with the aim of facilitating the interpretation of the interaction, a series of simple effect analyses were performed with Bonferroni correction applied to adjust for multiple comparisons. The first simple effects analysis evaluated the effect of event location while keeping the other conditions constant. This analysis showed that, in all cases, placing the event before a city led to better comprehension compared to after a city (*p*s < 0.05). The differences ranged from 13% and 27%. Furthermore, no significant differences were observed between drivers and non-drivers under any condition *(ps* > 0.05). Finally, a simple effects analysis was conducted to explore the effect of arrow function. The results showed that when the arrow was explicit, comprehension was higher than when it was generic (*p*s < 0.05), with differences ranging from 15% to 22%. It is worth noting that this was the case in all conditions, except for the non-driver group, when the event was located before a city. In that case, the difference did not reach statistical significance (*p* > 0.05). The remaining interactions involving the driving license factor were not significant, *Fs* < 3.48, *p*s > 0.05.

## 4. Discussion

The main objective of the present study was to compare the comprehension of different designs of VMS messages between drivers and non-drivers. Message design was manipulated across three factors. Landmark order (top-down vs. bottom-up) involved the vertical arrangement of city names, either from the nearest to the farthest location or in reverse, simulating the spatial flow of the route. Event location (before vs. after) indicated whether the described traffic event was situated before or after a designated reference point. Arrow function (generic vs. explicit) concerned the visual structure of the message. In the generic version, the event pictogram appeared within the alphanumeric column, accompanied by a general upward arrow positioned on the left side of the display. In the explicit version, the pictogram was placed on the left, and a smaller directional arrow was displayed next to each city name in the alphanumeric column. Results indicated that comprehension was lowest for both drivers and non-drivers when messages featured a top-down landmark order combined with a generic arrow. The remaining conditions were comparatively successful for both groups. However, unlike non-drivers, drivers exhibited a clear hierarchy in comprehension. The most effective combination was a bottom-up landmark order with an explicit arrow, followed by bottom-up with a generic arrow, and then top-down with an explicit arrow. In contrast, non-drivers showed no significant differences in comprehension across these combinations.

The results also revealed a significant interaction between arrow function, landmark order, and driving license status. This interaction was driven by the fact that, for drivers, comprehension was higher when the arrow was explicit compared to when it was generic, regardless of whether the event occurred before or after the city. In contrast, for non-drivers, this difference emerged only when the event was located after the city, and not when it was located before.

From a semantic perspective, meaning—i.e., the content of the premise—plays a key role in reasoning (Johnson-Laird, 2006). It is expected that individuals draw on both deductive and inductive inferences during the reasoning process, with inductive reasoning potentially supporting the interpretation of ambiguous or unclear elements based on prior experience or knowledge. In particular, the interpretation of the arrow (a deictic element) will depend on its position, shape, and direction in relation to the rest of the configuration (Kurata & Egenhofer, 2005, 2008; Tversky et al., 2000), and this, in turn, will influence the overall meaning of the message. We observed that the group of drivers exceeded 80% correct responses when the messages displayed an explicit arrow that was oriented upward (bottom-up order), making it stand out from other combinations. The adaptation observed in drivers (as opposed to non-drivers) parallels the findings of Hernando et al. (2022a): Dutch drivers, who were familiar with VMS capable of displaying diagonal arrows, demonstrated significantly better comprehension of horizontally arranged messages compared to drivers of other nationalities. It is important to understand how prior experience with a particular design, or with the use of elements involved in complex traffic messages (such as pictograms, arrows, etc.), can influence the comprehension of other signs. This, in turn, is linked to the role of familiarity, defined as an ergonomic principle related to prior experience that affects the comprehension of simple signs (Shinar et al., 2003). However, further research is needed in the field of complex signage to examine the effect of ergonomic principles on the overall comprehension of messages.

On the other hand, it is worth noting that the pattern of results for non-drivers was similar to that of drivers in response to certain message designs. For example, the combination that proved to be the worst for drivers (top-down order and generic arrow) was also the worst for non-drivers. The differences between this combination and the rest were the largest observed in the present study, with values ranging between 30% and 40%. In fact, the combination of top-down order, generic arrow, and event located after a city did not reach 24% correct responses in either group. These similarities in comprehension patterns may be linked to the general nature of reasoning and the multiple effects that influence the process. At the same time, this remains compatible with the idea that the construction of the PMM (preferred mental model), as well as any possible modifications made to it (Jahn et al., 2007; Oberauer & Wilhelm, 2000; Ragni & Knauff, 2013), is shaped by prior knowledge. This prior knowledge would guide reasoning—through inductive inferences—toward a particular possibility in each case.

All in all, the results align with the construction of incremental mental models by both drivers and non-drivers, in which ‘before’ locations are more easily comprehended than ‘after,’ consistent with bottom-up temporal preferences (Fuhrman & Boroditsky, 2010; Rinaldi et al., 2018) and with assumptions about model construction (Oberauer & Wilhelm, 2000; Ragni & Knauff, 2013). This pattern aligns with cognitive accounts of orienting and attention (Posner, 1980; Posner & Dehaene, 1994) and with the interaction between relative and intrinsic frames of reference in sign interpretation (Johnson-Laird, 2006; Levinson, 2003). Language itself reflects similar tendencies, as generic metaphors also represent the future as being in front of the ego (Núñez & Sweetser, 2006). Taken together, these findings suggest a shared bottom-up spatial preference that favors the comprehension of vertical layouts in VMS across both drivers and non-drivers.

### 4.1. Practical Implications

As in Hernando et al. (2022a, 2022b), it has once again been confirmed that the factors associated with message design (event location, arrow function and landmark order) have played a key role in message comprehension, among the non-driver group as well. Moreover, our results suggest that the cognitive processes involved in message comprehension are of a general nature (not specific to or exclusively associated with driving). This observation is important because, in the move toward harmonizing traffic signs, research and consideration should not be limited to drivers alone, but should also include all active agents who may be part of the driving environment—such as pedestrians, cyclists, and scooter users. These agents must also be able to understand and respond to the information conveyed by the message. On the other hand, there is strong support in the wayfinding and signage literature demonstrating that arrows and pictograms used in airports, metro stations, railway stations, museums, and ports, as well as on roads, share common design features and serve similar functions in guiding users effectively (Harding et al., 2017; Sign Research Foundation, 2016; Majid & Ismail, 2016; International Organization for Standardization, 2023).

Although this study used only Spanish place names, previous research suggests that comprehension of VMS combinations is comparable across several European languages (Hernando et al., 2022a). Nevertheless, because writing systems and reading conventions may influence how road signs are processed, future studies should examine whether the effects observed here generalize to a broader range of languages and scripts. Information needs are evolving, and signage must be capable of adapting to this increasingly globalized and diverse traffic scenario. Continuing to explore the key factors involved in sign comprehension contributes to progress toward updated and effective traffic signs.

### 4.2. Limitations and Future Research Directions

This study presents certain limitations that should be taken into account. The main limitation concerns the sample, which consisted predominantly of young individuals from a single nationality (Spanish). This limitation is relative, as prior research has already assessed cross-cultural generalizability with drivers of different ages (e.g., Lucas-Alba et al., 2011; Hernando et al., 2022a, 2022b). However, future studies should include drivers and non-drivers of different ages, and from different nationalities to broaden the generalizability of the results. Moreover, participants were recruited through convenience sampling from among our own students and university staff, which led to an unbalanced distribution of sex across groups. This imbalance reflects the recruitment context of a psychology program in which female students are overrepresented, particularly among those without a driving license. Such uneven distribution may have influenced the findings and therefore constrains the generalizability of the results. Considering the possibility of gender imbalances in spatial cognition (Coluccia & Louse, 2004), future research should aim to include a more even representation of sex across groups, in order to determine whether the observed effects hold when the potential influence of sex is explicitly controlled.

A further limitation is that group membership was defined by formal training in traffic regulations rather than practical driving experience. Future studies should examine how different levels of actual driving experience may affect traffic sign comprehension. In addition, although response speed was emphasized during the task to approximate naturalistic driving conditions, the study did not analyze response times. Future work should incorporate this measure, as it would provide valuable complementary insights once high levels of accuracy are achieved.

Another limitation is that only Spanish place names were used in this study. Because reading order and spatial processing can be influenced by the conventions of different writing systems (e.g., left-to-right, right-to-left, or logographic scripts), the present findings may not fully generalize across languages. Subsequent studies should therefore examine whether the observed effects are consistent when place names are presented in languages and writing systems with different reading conventions.

Future research should also incorporate technological tools such as driving simulators or eye-tracking recordings. Additionally, it would be valuable to measure and include more variables related to prior knowledge and/or driving experience in the explanatory model, as well as working memory and cognitive load measures, in order to explore their role in the inferential process involved in interpreting complex traffic messages. This would help deepen our understanding of the reasoning mechanisms that may explain the differences observed between the two groups (drivers and non-drivers).

All of these improvements would contribute to the gathering of richer information and the achievement of a more integrated and comprehensive understanding of reasoning processes in a more realistic environment.

## 5. Conclusions

The present study demonstrates that comprehension of variable message signs depends critically on specific design features and on whether or not the participant possesses a driver’s license. Across both groups, the combination of a top-down order with a generic arrow consistently yielded the lowest comprehension rates compared to other combinations. This result indicates that the G1c template from the 1968 Convention, when adapted to VMS in this way, is not intuitive for either drivers or non-drivers.

Participants with a driver’s license showed a clear hierarchy in comprehension: the bottom-up order with an explicit arrow produced the highest accuracy, better than the top-down/explicit combination and much better than the bottom-up/generic combination (see also Hernando et al., 2022b). In contrast, participants without a driver’s license did not establish such a hierarchy; they perceived all viable designs more uniformly, with significant differences emerging only when comparing bottom-up/explicit to top-down/explicit arrangements. These findings suggest that driver’s license status selectively enhances sensitivity to certain design cues, particularly explicit directional arrows presented in a bottom-up order.

Independent of driver’s license status, comprehension was consistently higher when the event was located before a city rather than after it. Additionally, explicit arrows were generally superior to generic ones, increasing comprehension across most conditions.

Taken together, these findings show that design factors do not merely influence comprehension in a generic way but interact with driver’s license status to shape how information is processed. The evidence suggests that harmonized VMS design should prioritize bottom-up arrangements, explicit arrows, and “before” event locations to maximize comprehension across diverse road users. At the same time, the fact that participants without a driver’s license understood several viable designs at comparable levels underscores that comprehension is not exclusively dependent on driving expertise but reflects broader reasoning processes shared across populations.

From a practical perspective, these results provide an empirically grounded basis for updating and harmonizing VMS standards. The data identify specific combinations of factors that optimize comprehension, offering concrete guidance for road authorities seeking to design universally intelligible messages.

In sum, our results show that key message design factors strongly influence comprehension across all road users, not just drivers, underscoring the need for updated signage standards that address an increasingly diverse and global traffic environment. Road traffic is a complex system that demands the utmost attention. The number of participants in this system—especially in densely populated urban areas—is steadily increasing, within a society driven by mobility through a wide range of options, from walking to buses, including bicycles, scooters, motorcycles, Segway, cars, and more. Moreover, the need to move, to get somewhere, to orient oneself toward a destination is not limited to motorized traffic. At some point (as the literature on wayfinding and signage seems to suggest), the cognitive resources required to reach a destination are shared by all humans in motion. Perhaps this common foundation explored in the present study could encourage the sharing and integration of what we already know—and what we can further discover—about what helps us understand where we are going.

## Data Availability

The raw data supporting the conclusions of this article will be made available by the authors on request.

## Abbreviations

The following abbreviations are used in this manuscript:

ANOVA	Analysis of Variance
MM	Mental Model
PMM	Preferred Mental Model
UNECE	United Nations Economic Commission for Europe
VMS	Variable Message Signs

## Appendix A

**Table A1:** Experimental design and messages included in this study.

Event Location	Complementary Message		Arrow Function	Landmark Order	Target Message
Before	Present		Explicit	Top-down	
				Bottom-up	
			Generic	Top-down	
				Bottom-up	
	Absent		Explicit	Top-down	
				Bottom-up	
			Generic	Top-down	
				Bottom-up	
After	Present		Explicit	Top-down	
				Bottom-up	
			Generic	Top-down	
				Bottom-up	
	Absent		Explicit	Top-down	
				Bottom-up	
			Generic	Top-down	
				Bottom-up	

Note: The experimental design summarizes the 16 trials that were included in the statistical analyses of this study.
